# Cardiovascular disease competes with breast cancer as the leading cause of death for older females diagnosed with breast cancer: a retrospective cohort study

**DOI:** 10.1186/bcr2901

**Published:** 2011-06-20

**Authors:** Jennifer L Patnaik, Tim Byers, Carolyn DiGuiseppi, Dana Dabelea, Thomas D Denberg

**Affiliations:** 1 Department of Epidemiology, Colorado School of Public Health, University of Colorado Denver, 13001 E. 17th Place, Aurora, CO 80045, USA; 2 Department of Quality and Patient Safety, Atrius Health and Harvard Vanguard Medical Associates, 275 Grove Street, Newton, MA 02466, USA

## Abstract

**Introduction:**

Many women who survive breast cancer die of causes unrelated to their cancer diagnosis. This study was undertaken to assess factors that are related to breast cancer mortality versus mortality from other causes and to describe the leading causes of death among older women diagnosed with breast cancer.

**Methods:**

Women diagnosed with breast cancer at age 66 or older between 1992 and 2000 were identified in the Surveillance, Epidemiology and End Results-Medicare linked database and followed through the end of 2005.

**Results:**

A total of 63,566 women diagnosed with breast cancer met the inclusion criteria and were followed for a median of approximately nine years. Almost one-half (48.7%) were alive at the end of follow-up. Ages and comorbidities at the time of diagnosis had the largest effects on mortality from other causes, while tumor stage, tumor grade, estrogen receptor status, age and comorbidities at the time of diagnosis all had effects on breast cancer-specific mortality. Fully adjusted relative hazards of the effects of comorbidities on breast cancer-specific mortality were 1.24 (95% confidence interval (95% CI) 1.13 to 1.26) for cardiovascular disease, 1.13 (95% CI 1.13 to 1.26) for previous cancer, 1.13 (95% CI 1.05 to 1.22) for chronic obstructive pulmonary disease and 1.10 (95% CI 1.03 to 1.16) for diabetes. Among the total study population, cardiovascular disease was the primary cause of death in the study population (15.9% (95% CI 15.6 to 16.2)), followed closely by breast cancer (15.1% (95% CI 14.8 to 15.4)).

**Conclusions:**

Comorbid conditions contribute importantly to both total mortality and breast cancer-specific mortality among breast cancer survivors. Attention to reducing the risk of cardiovascular disease should be a priority for the long-term care of women following the diagnosis and treatment of breast cancer.

## Introduction

Mortality rates for breast cancer have declined since 1990. In 2006, there were about 2.5 million women in the United States who had survived breast cancer (diagnosed at any point prior to 1 January 2006) [1]. The survivorship of breast cancer patients depends on many factors, including age at diagnosis, tumor stage, tumor grade, estrogen receptor (ER) status, progesterone receptor status, socioeconomic factors and other non-cancer-related clinical conditions such as preexisting health status, functional status and social connections [2-4]. Research has shown that other preexisting chronic conditions, or comorbidities, also have a significant effect on the overall survival of breast cancer patients [5-7]. The current study assesses the effects of comorbidities on outcomes of breast cancer mortality and mortality from other causes among breast cancer survivors ages 66 and older.

The most commonly used outcome when conducting epidemiologic research on cancer survivorship is all-cause mortality, which, among individuals with breast cancer, is the sum of breast cancer-specific mortality and mortality from other causes. Although assigning a single cause of death is difficult and can be somewhat arbitrary, assessing outcomes for both breast cancer-specific mortality and mortality from other causes can assist healthcare providers and patients with breast cancer in assessing prognostic indicators and other decisions made in primary care. This study describes the effect of select characteristics, including comorbid conditions, on breast cancer-specific mortality compared to mortality from other causes and explores leading causes of death among breast cancer survivors.

## Materials and methods

### Data source

The Surveillance, Epidemiology and End Results (SEER)-Medicare linked database was utilized for this analysis. This database is a linkage of records from SEER, a large cancer registry, and Medicare, the national healthcare system for older persons. The SEER registry is designed to track primary cancer incidence and survival in the United States. Although not all US states report cancer cases to the SEER program, it is designed to be representative of data from the whole country. SEER participation has expanded greatly since its inception. While initially capturing approximately 10% of the US population in 1973, the SEER program has grown to comprise 26% of the US population currently [8]. The population covered by SEER is comparable to the general US population with regard to measures of poverty and education. However, the SEER registry population represents a somewhat more urban population and has a higher proportion of foreign-born persons than are found in the general US population. The compilers of the SEER registry continually monitor and evaluate their data to ensure high quality. The program's standard for registry completeness for the incidence of cancer cases is 98% [9]. The SEER registry collects information on gender, race, and dates of birth and death, cause of death, as well as tumor-specific information such as diagnosis date, tumor stage at diagnosis, tumor grade, ER and progesterone receptor status, and surgery and/or radiation treatments within four months after cancer diagnosis.

Medicare is a US system of federally funded health insurance for persons ages 65 years and older. Medicare part A covers hospital, skilled-nursing facility, hospice and some home health care services. Medicare part B covers physician and outpatient services. Virtually all beneficiaries receive part A, and 96% of beneficiaries choose to pay a monthly premium for part B. Medicare patients can be enrolled in either a fee-for-service (FFS) or health maintenance organization (HMO) plan. Claims files are not available for the 17% of Medicare recipients who are in an HMO plan [9]. Medicare data include demographic information and surgical and radiation treatment information that are already in the SEER database, as well as information on comorbid conditions and chemotherapy treatment. The SEER database has a unique code that does not include identifying information; therefore, the research that we conducted was exempt from institutional review board approval by the Colorado Multiple Institutional Review Board.

### Study population

Women diagnosed with primary cancers of the breast at age 66 years or older and entered into the SEER data set from 1 January 1992 through 31 December 2000 were eligible for inclusion into our study. Women ages 65 years old were not included to allow for a one-year time period following Medicare enrollment during which comorbidities could be recorded in claims files. Women were excluded if they (1) lacked full insurance coverage under both Medicare part A and part B, (2) were enrolled in a Medicare HMO, (3) had an unknown month of cancer diagnosis, (4) had the same month of diagnosis and death, (5) had an unknown cause of death, and/or (6) had records considered to have unreliable Medicare coding for a comorbid condition (most commonly bills that were not encoded by a clinician for a specific reason for the visit). These exclusion criteria ensured that claims files were available for accurate detection of comorbidities and calculation of survival times from diagnosis to death.

A total of 96,954 women ages 66 years and older diagnosed with malignant breast cancer between 1992 and 2000 were potentially eligible for inclusion in this study. Just over one-third (33,388, or 34.4%) were excluded for the following reasons: being in a Medicare HMO (22.8%), unreliable diagnosis coding (4.0%), not having both Medicare parts A and B insurance coverage (5.5%), month and year of death the same as the diagnosis (2.6%), unknown month of diagnosis (0.7%) and/or unknown cause of death (0.5%).

### Measurement of treatment and comorbidities

Medicare files were searched for diagnostic and procedural codes related to the comorbidities and treatments of interest [10]. Codes for diagnoses and procedures were derived from the *International Classification of Diseases, Ninth Revision, Clinical Modification *(ICD-9-CM) [11], the Health Care Financing Administration Common Procedure Coding System and revenue center codes [12]. Medicare files for claims from inpatient, outpatient and physician visits were utilized.

In addition to SEER fields of surgery and radiation treatment within four months after breast cancer diagnosis, Medicare files were searched for chemotherapy and radiation codes. Patients with at least one chemotherapy code within six months after cancer diagnosis and patients with at least one radiation therapy code within nine months after cancer diagnosis were categorized as receiving those respective therapies. Data on hormonal therapy are not available in the SEER-Medicare database and therefore were not included in our analysis. Specific codes used to identify chemotherapy and radiation treatment are listed in Additional file 1.

Comorbidities included in this study are those 19 conditions that were found to affect survival by Charlson *et al*. [13,14] and adapted by Deyo *et al*. [15] and Klabunde *et al*. [16] to be used with administrative claims data. Rather than using the combined Charlson Comorbidity Index, the current study assesses the individual effects of each of the comorbidities included in the Charlson Index. The Medicare files were searched for comorbidity diagnoses appearing during the time periods one year before and 30 days after the cancer diagnosis. This longer period before cancer diagnosis and the short period after cancer diagnosis allows for substantial time to identify diagnoses representing comorbidities without capturing conditions that may result from the cancer treatment. Diagnostic coding for comorbidities was made only if the comorbidity condition was included in Medicare inpatient files or if the condition had been coded at least twice at least 30 days apart in either the outpatient or physician files. The comorbid condition of previous cancer was determined from the SEER database.

For this analysis, we combined Charlson categories that were separated by severity (for example, diabetes and diabetes with complications) and further grouped conditions of myocardial infarction, congestive heart failure, peripheral vascular disease and cerebrovascular disease into one category of cardiovascular disease (CVD). Women without any of the 19 conditions comprised the group "No comorbidities." Only the four most common comorbid conditions within the Charlson index, previous cancer, CVD, chronic obstructive pulmonary disease (COPD) and diabetes, are included in the current study.

### Underlying cause of death

The SEER registry uses US state death certificates to obtain information on the underlying cause of death encoded with the ICD-9 diagnostic codes. The underlying cause of death according to the ICD-9 diagnostic codes is defined as "the disease or injury that initiated the train of morbid events leading directly to death or the circumstances of the accident or violence that produced the injury" [17]. In the current study, deaths were attributed to either breast cancer or other causes. Deaths due to cancers other than breast cancer are included in the other-cause mortality group.

### Statistical analysis

The study population was categorized into three study groups: (1) women who were alive at the end of the study period, (2) women who died as a result of breast cancer-specific reasons during the study period and (3) women who died as a result of other causes. For analyses involving breast cancer-specific mortality, deaths from other causes were censored at time of death and *vice versa*. Since the SEER registry does not include the specific diagnosis date or the specific death date, the 15th of the month was arbitrarily assigned to the reported month and year. The study population was followed through the end of 2005.

Descriptive statistics of the three study groups are presented. Cox proportional hazards models were used to calculate relative hazards of breast cancer mortality and other-cause mortality for population characteristics adjusted for age and fully adjusted for age and other characteristics. Causes of death were grouped into the three leading causes: breast cancer, other cancer and CVD. All other causes of death were grouped into the category "Other." Proportional distributions of cumulative causes of death were explored according to age group (66 to 74 years, 75 to 84 years and 85 years and older) and cancer stage (stage I, stage II and stages III and IV), as well as by duration of follow-up. Missing or unknown data were grouped into their own categories and were included in Cox proportional hazards models. Two approaches were used to assess whether missing predictors had an influence on the main findings: (1) records with missing or unknown values for any variables of interest were excluded from the analyses, and (2) the R package NestedCohort [18] was used to weight records with known values regarding age, race/ethnicity and death status to account for records with missing data. Accounting for missing or unknown data did not influence the results. SAS version 9.1 software (SAS Institute, Cary, NC, USA) was used for analysis.

## Results

A total of 63,566 women met the study inclusion criteria. Among these women, 32,594 individuals (51.3%) died during the study period: 15.1% (95% confidence interval (95% CI) 14.8 to 15.4) of the study population died as a result of breast cancer and 36.2% (95% CI 35.5 to 36.3) died as a result of other causes. The median follow-up time was 105 months (range 1 to 167 months), and the median age at death was 83 years (range 66 to 110 years). Women with higher tumor stages and grades and ER-negative status were more likely to die as a result of breast cancer during the study period. Older women were much more likely to die as a result of other causes (Table 1).

**Table 1:** Prevalence of population characteristics by mortality status among breast cancer patients ages 66 years and older, SEER-Medicare data from 1992 to 2000^a^

Characteristics	Alive	Breast cancer deaths	Other-cause deaths
Total, *n *(%)	30,972 (48.7%)	9,608 (15.1%)	22,986 (36.2%)
Age, years, *n *(%)			
85+	18,054 (63.0%)	4,021 (14.0%)	6,572 (22.9%)
75 to 84	11,543 (43.9%)	3,900 (14.8%)	10,824 (41.2%)
66 to 74	1,375 (15.9%)	1,687 (19.5%)	5,590 (64.6%)
Race/ethnicity, *n *(%)			
White	27,224 (48.9%)	8,170 (14.7%)	20,329 (36.5%)
Black	1,481 (38.3%)	889 (23.0%)	1,501 (38.8%)
Hispanic	966 (50.8%)	324 (17.0%)	613 (32.2%)
Other/unknown	1,301 (62.9%)	225 (10.9%)	543 (26.2%)
Stage, *n *(%)			
I	19,447 (59.3%)	1,735 (5.3%)	11,597 (35.4%)
II	9,001 (44.1%)	3,546 (17.4%)	7,856 (38.5%)
III and IV	911 (15.4%)	3,296 (55.6%)	1,724 (29.1%)
Unknown	1,613 (36.2%)	1,031 (23.2%)	1,809 (40.6%)
Grade, *n *(%)			
1	6,410 (61.3%)	497 (4.8%)	3,546 (33.9%)
2	11,966 (53.0%)	2,556 (11.3%)	8,052 (35.7%)
3 and 4	7,121 (42.6%)	3,917 (23.4%)	5,694 (34.0%)
Unknown	5,475 (39.6%)	2,638 (19.1%)	5,694 (41.2%)
ER status, *n *(%)			
Positive	21,388 (52.5%)	4,784 (11.7%)	14,568 (35.8%)
Negative	3,471 (43.8%)	2,006 (25.3%)	2,451 (30.9%)
Unknown/other	6,113 (41.0%)	2,818 (18.9%)	5,967 (40.0%)
Surgery, *n *(%)			
Yes	30,506 (51.1%)	7,658 (12.8%)	21,553 (36.1%)
No	152 (6.4%)	1,297 (54.7%)	921 (38.9%)
Unknown	314 (21.2%)	653 (44.2%)	512 (34.6%)
Chemotherapy, *n *(%)			
Yes	4,709 (50.0%)	2,724 (29.0%)	1,975 (21.0%)
No	26,263 (48.5%)	6,884 (12.7%)	21,011 (38.8%)
Radiation therapy, *n *(%)			
Yes	13,350 (61.5%)	2,798 (12.9%)	5,556 (25.6%)
No	17,622 (42.1%)	6,810 (16.3%)	17,430 (41.6%)
Comorbidity, *n *(%)			
None	21,017 (56.7%)	5,500 (14.8%)	10,544 (28.4%)
Previous cancer	4,280 (41.4%)	1,533 (14.8%)	4,534 (43.8%)
Cardiovascular disease	1,955 (24.1%)	1,353 (16.7%)	4,794 (59.2%)
COPD	1,886 (33.6%)	798 (14.2%)	2,927 (52.2%)
Diabetes	3,000 (36.4%)	1,308 (15.9%)	3,939 (47.8%)

^a^SEER: Surveillance, Epidemiology and End Results; ER: estrogen receptor; COPD: chronic obstructive pulmonary disease.

Prevalence estimates at the time of diagnosis of breast cancer were 16.3% for previous cancer, 13.0% for diabetes, 12.8% for CVD and 8.8% for COPD, and the prevalence estimates were higher for older women. Women with no comorbidities were more likely than women with comorbidities to be alive at the end of the study period (Table 1). In the overall study population, 36.2% of women died as a result of causes other than breast cancer. Women with the following comorbidities were more likely to die as a result of other causes: CVD (59.2%), COPD (52.2%), diabetes (47.8%) and previous cancer (43.8%).

The age-adjusted relative hazards of dying as a result of breast cancer and other causes were greater among women with higher tumor stages and grades, black women, women with ER-negative status and women with any of the select comorbid conditions (Table 2). After adjustment for age, tumor stage and grade, ER status, treatments and all other comorbid conditions, the relative hazards of dying were different for breast cancer-specific mortality versus other-cause mortality. Age affected both types of mortality, but relative hazards were much higher when other-cause mortality was the outcome. Tumor characteristics of stage and grade as well as ER status greatly affected breast cancer mortality, whereas only tumor stage affected other-cause mortality, and to a much lesser extent than that associated with breast cancer mortality. Relative hazards for comorbidities were more highly associated with other-cause mortality, although all comorbid conditions also significantly affected breast cancer mortality. Many of the changes in the relative hazards from the age-adjusted model to the fully adjusted model are due to adjustments for tumor stage among breast cancer deaths and for treatment for both breast cancer deaths and other-cause deaths.

**Table 2:** Age-adjusted and fully adjusted relative hazards of breast cancer mortality and other-cause mortality among breast cancer patients ages 66 years and older, SEER-Medicare data from 1992 to 2000^a^

	Breast cancer deaths		Other-cause deaths	
Characteristics	Age-adjusted model	**Fully adjusted model**^**b**^	Age-adjusted model	**Fully adjusted model**^**b**^
Age, years	Crude		Crude	
66 to 74	Reference	Reference	Reference	Reference
75 to 84	1.21 (1.16 to 1.27)	1.20 (1.14 to 1.26)	2.28 (2.21 to 2.34)	2.04 (1.98 to 2.10)
85+	2.23 (2.10 to 2.36)	1.67 (1.57 to 1.78)	6.08 (5.86 to 6.31)	4.63 (4.46 to 4.82)
Race/ethnicity				
White	Reference	Reference	Reference	Reference
Black	1.79 (1.67 to 1.92)	1.12 (1.05 to 1.21)	1.29 (1.23 to 1.36)	1.05 (0.99 to 1.10)
Hispanic	1.21 (1.08 to 1.35)	0.96 (0.86 to 1.08)	0.97 (0.89 to 1.05)	0.87 (0.81 to 0.95)
Other/unknown	0.73 (0.64 to 0.83)	0.76 (0.66 to 0.87)	0.75 (0.68 to 0.81)	0.73 (0.67 to 0.80)
Stage				
I	Reference	Reference	Reference	Reference
II	3.72 (3.51 to 3.94)	3.05 (2.88 to 3.24)	1.26 (1.23 to 1.30)	1.24 (1.20 to 1.27)
III and IV	19.9 (18.8 to 21.1)	11.50 (10.8 to 12.3)	1.76 (1.67 to 1.85)	1.65 (1.56 to 1.74)
Unknown	5.38 (4.98 to 5.81)	2.88 (2.64 to 3.13)	1.37 (1.30 to 1.44)	1.03 (0.97 to 1.09)
Grade				
1	Reference	Reference	Reference	Reference
2	2.45 (2.22 to 2.70)	1.76 (1.60 to 1.94)	1.07 (1.03 to 1.11)	1.04 (1.00 to 1.08)
3 and 4	5.64 (5.14 to 6.20)	2.63 (2.39 to 2.90)	1.18 (1.13 to 1.23)	1.09 (1.05 to 1.14)
Unknown	4.34 (3.94 to 4.78)	1.93 (1.75 to 2.13)	1.21 (1.16 to 1.27)	1.06 (1.01 to 1.11)
ER status				
Positive	Reference	Reference	Reference	Reference
Negative	2.48 (2.35 to 2.61)	1.71 (1.62 to 1.81)	1.05 (1.01 to 1.10)	1.03 (0.99 to 1.08)
Unknown/other	1.78 (1.70 to 1.87)	1.24 (1.18 to 1.31)	1.22 (1.18 to 1.26)	1.08 (1.05 to 1.12)
Comorbidity				
None	Reference	Reference	Reference	Reference
Previous cancer	1.10 (1.03 to 1.16)	1.20 (1.13 to 1.26)	1.68 (1.63 to 1.74)	1.29 (1.25 to 1.34)
Cardiovascular disease	1.44 (1.36 to 1.53)	1.24 (1.17 to 1.32)	2.83 (2.73 to 2.94)	1.87 (1.80 to 1.93)
COPD	1.20 (1.12 to 1.30)	1.13 (1.05 to 1.22)	2.66 (2.56 to 2.78)	1.71 (1.65 to 1.78)
Diabetes	1.31 (1.24 to 1.40)	1.10 (1.03 to 1.16)	2.37 (2.28 to 2.46)	1.57 (1.52 to 1.63)

^a^SEER: Surveillance, Epidemiology and End Results; ER: estrogen receptor; COPD: chronic obstructive pulmonary disease. ^b^Adjusted for age, race/ethnicity, stage, grade, ER status, surgery, radiation, chemotherapy and all comorbidities. Data are expresses as relative hazards (95% confidence intervals).

**Figure 1:**
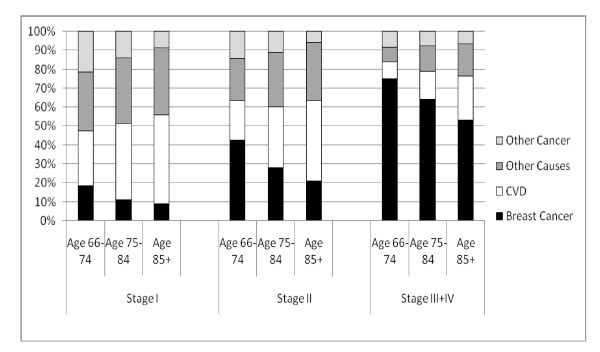
**Proportional distribution of leading causes of death among breast cancer patients ages 66 years and older by age at diagnosis and by stage of disease from 1 January 1992 through 31 December 2000**. CVD: cardiovascular disease.

The distribution of causes of death varied by age and tumor stage (Figure 1). Women diagnosed with higher-stage cancers were much more likely to die as a result of breast cancer than from other causes. Other causes of death were more likely among women diagnosed with stages I and II cancers. At all ages, women diagnosed with stage I breast cancer were more likely to die as a result of CVD than from breast cancer. CVD was also the leading cause of death for women diagnosed with stage II breast cancer who were ages 85 years and older (42.2%) and 75 to 84 years of age (32.2%). Within all cancer stages, as women aged, there was a decrease in the proportion of all deaths that were due to breast cancer.

**Figure 2:**
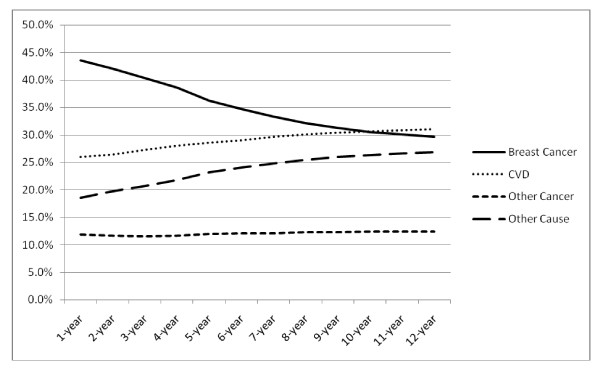
**Proportional distribution of cumulative leading causes of death by time since breast cancer diagnosis**. CVD: cardiovascular disease.

The proportional distribution of cumulative causes of death was dependent on follow-up time. In the first 5 to 10 years following breast cancer diagnosis, breast cancer was the cumulative primary cause of death. With longer follow-up, CVD became the cumulative primary cause of death (Figure 2). The proportion of deaths due to other causes also increased as follow-up increased. The primary causes of death in this category included deaths due to COPD (3.8% of all deaths), pneumonia (2.8%), Alzheimer's disease (2.2%) and diabetes (2.2%).

## Discussion

Older female Medicare enrollees were more than twice as likely to die as a result of diseases other than breast cancer (36.2% versus 15.1%). Characteristics associated with dying from other causes included older age, higher tumor stage and the presence of comorbidities, whereas characteristics associated with dying as a result of breast cancer included higher tumor-related stage and grade, ER-negative status and the presence of comorbidities. The finding that comorbidities had a significant effect on breast cancer-specific mortality is new and of particular interest, although relative hazards were significantly lower than those for other-cause mortality.

CVD was the leading cause of death in the study population (15.9%), closely followed by breast cancer (15.1%). CVD was the primary cause of death for women of all ages with stage I cancer diagnoses and for women in the two older age groups with stage II cancers. Within each cancer stage group, as women aged, they were more likely to die as a result of CVD and "other" causes and less likely to die as a result of breast cancer, other cancers or COPD. These findings are likely due to the facts that older women are more likely to have comorbidities [19] and that women with comorbidities are more likely to die as a result of causes other than breast cancer. This same age pattern is also seen in nationwide US vital statistics for the general population [20]. As expected, our study population was more likely to die as a result of cancer and less likely to die as a result of heart disease than older females in the general study population.

Our study comprised a long follow-up period (median 105 months). Over time, as women survive their breast cancer, they become less likely to die as a result of their cancer diagnosis. Still, even within the first five years of follow-up, CVD remains the leading cause of death for women of all ages with stage I breast cancers (data not shown).

Of those women who died as a result of CVD in our study, only 25.5% were also categorized as having CVD as a comorbid condition at the time of their breast cancer diagnosis. Conversely, of those women who had CVD as a comorbid condition at the time of their breast cancer diagnosis and who died during the study period, 41.9% died as a result of CVD. These percentages are low for several reasons. Most importantly, there was a long period of follow-up in this study, which allows for a substantial amount of time for women to develop CVD subsequent to our detection of the condition at the time of the breast cancer diagnosis. Chemotherapy has been shown to be associated with long-term cardiac toxicity in breast cancer patients, particularly in older patients [21]. In addition, there is likely to be underdiagnosis of CVD and/or lack of reporting in the Medicare record. Underdiagnosis and undertreatment of CVD may be important issues for women diagnosed with cancer, as their cancer diagnosis can be perceived by both the patient and her caregivers as the overriding medical priority.

We found four studies that have assessed the effect of patient characteristics apart from tumor characteristics on cause-specific breast cancer mortality. Two of these studies determined that patients over 65 or 70 years of age were more likely than younger patients to die as a result of other causes [22,23]. Another study found that women with three or more comorbid conditions had a 20-fold higher rate of non-breast cancer mortality and a fourfold higher rate of all-cause mortality than did women diagnosed with breast cancer without comorbidities [24]. The third study assessed the role of comorbidity in explaining the shorter survival of black breast cancer patients and found that black women diagnosed with breast cancer were more likely to die as a result of causes other than breast cancer [25]. We also found that black women were slightly more likely to die as a result of other causes, but, after adjustment for cofactors, we found that black women had survival rates similar to those of white women.

In the current study, we detected a positive association between comorbidities and breast cancer-specific mortality. We are not aware of a previous study that has highlighted this relationship. There could be several potential explanations for these associations. Women with comorbidities are less likely to be treated or may be more likely to experience adverse effects of therapy and not complete their treatment regimens. Therefore, women with comorbidities could be more likely to die as a result of breast cancer. In addition, there may be misclassification in cause-of-death coding, and women with metastatic disease may be more likely to have their cancer listed as the underlying cause of death when another factor may have been the true primary cause. A surprising finding in our study was the significant association between tumor stage and mortality as a result of other causes. Again, it is not clear whether these findings indicate that women with higher-stage tumors are at higher risk of non-cancer-related mortality and women with comorbidities are at higher risk of breast cancer-specific mortality or whether these findings are due to misclassification in cause-of-death coding.

There can be considerable controversy surrounding the determination of a specific cause of death, so our study is limited by the validity of death coding. However, mortality data have been regulated for over 50 years by the World Health Organization's International Classification of Diseases [26]. Standard forms and model procedures are used for the collection of data and registration of events. One of the goals of this standardization is to have death certificates that are comparable both internationally and nationally. Nonetheless, the validity of cause-of-death determination is questionable and has been found to differ across cancer types, time periods and ages at death [27]. In addition, data from the Framingham Heart Study [28] have indicated that heart disease is overestimated as a cause of death in the general population. If this is true in our study population, then we cannot conclude that CVD is the primary cause of death, although the rates of CVD and breast cancer are likely very similar in this study population of older women diagnosed with breast cancer.

The current study is also limited by the fact that Medicare data were created for billing purposes and may not be optimal for clinical research. Recipients of Medicare may seek care outside their Medicare coverage, such as long-term care, oral prescriptions of tamoxifen or hormonal therapy. Treatments of both breast cancer and comorbid conditions have improved since the beginning of the period covered by our study. Information regarding medical history prior to enrolling in Medicare is also not available. The measurement of comorbidities does not consider the severity illness or the duration of diagnosis, and comorbidities that are present but not covered by Medicare would result in misclassification of comorbidity status. In addition, data on possible confounding factors, such as smoking, diet and functional status, are not available in the database.

A sizable portion (34%) of the eligible study participants were excluded from this study, mainly because they were enrolled in HMOs, for which Medicare files are not available. Differences do exist between these two Medicare healthcare delivery systems. Patients enrolled in HMOs are generally younger, healthier, diagnosed at an earlier stage and have better overall survival than FFS patients [9,29]. There is no reason to believe that the differences between HMO and FFS patients would bias the findings observed in our study, but our results are most generalizable to older adults who are FFS patients.

In addition, there are a number of records with missing or unknown values for tumor stage and grade as well as ER status. These missing covariates are related to the study outcomes. Women who died during the study period were more likely to have unknown tumor stage and grade, ER status and surgery status. Missing tumor characteristics (tumor stage and grade as well as ER status) do not appear to be related to the specific cause of death (categorized as "Breast cancer" or "Other cause"). However, women with unknown surgery status were more likely to die as a result of breast cancer. Although the missing data are not random, they are an intrinsic characteristic of surveillance databases. Analyses done to account for missing data did not influence the study findings or conclusions.

## Conclusions

Many older women diagnosed with breast cancer die as a result of comorbid conditions rather than from breast cancer. Other studies have found that comorbidities are associated with increased risk of mortality from other causes, and in our study we found that women with comorbidities were more likely to die as a result of breast cancer as well. We found that after 12 years of follow-up, older women diagnosed with breast cancer were almost equally likely to die as a result of breast cancer as they were to die as a result of CVD. Patient management rightfully focuses on cancer after diagnosis, but consideration of other existing comorbid conditions should also be integrated into patient management and recovery plans [30-32]. Especially among older breast cancer survivors, risk management of factors associated with CVD can help improve overall survival.

## Abbreviations

COPD: chronic obstructive pulmonary disease; CVD: cardiovascular disease; ER: estrogen receptor; FFS: fee for service; HMO: health maintenance organization; ICD: International Classification of Diseases; SEER: Surveillance, Epidemiology and End Results.

## Competing interests

The authors declare that they have no competing interests.

## Authors' contributions

All authors contributed to the conception, design and interpretation of the data; the preparation of the manuscript; and the final editing and approval of the manuscript. All authors read and approved the final manuscript.

## Supplementary Material

Additional file 1 **Codes used to identify chemotherapy and radiation treatment in Medicare files**. This file contains Medicare codes that were used to indicate chemotherapy and radiation treatment. Click here for file
