# Supplementary material for: Cardiovascular disease competes with breast cancer as the leading cause of death for older females diagnosed with breast cancer: a retrospective cohort study
Source: Breast Cancer Res. 2011 Jun 20;13(3):R64. doi: 10.1186/bcr2901 (PMC3218953; doi:10.1186/bcr2901)
Supplement: Additional file 1 — Codes used to identify chemotherapy and radiation treatment in Medicare files. This file contains Medicare codes that were used to indicate chemotherapy and radiation treatment. [file bcr2901-S1.DOCX]

**Additional file 1. Codes used to identify chemotherapy and radiation treatment in Medicare files.**

|  | **ICD-9 Diagnostic Codes** | **ICD-9 Procedure Codes** | **HCPCS Codes** | **Revenue Center Codes** |
| --- | --- | --- | --- | --- |
| **Chemotherapy** | V58.1  V66.2  V67 | 99.25 | 96400-96499  96500-96599  Q0083-Q0085  51720  J8510  J8520  J8521  J8530-J8999  J9000-J9999 | 331  332  335 |
| **Radiation** | V58.0  V66.1  V67.1 | 92.21-92.33  92.39 | 77401-77499  77520  77523  77750-77799  G0256  G0261 | 330  333  339 |
